# Effect of anodized zirconium implants on early osseointegration process in adult rats: a histological and histomorphometric study

**DOI:** 10.1007/s40204-019-00124-0

**Published:** 2019-11-22

**Authors:** María Florencia Tano de la Hoz, María Rosa Katunar, Ariel González, Andrea Gomez Sanchez, Alcira Ofelia Díaz, Silvia Ceré

**Affiliations:** 1 INTEMA, Applied Electrochemistry Division, National University of Mar del Plata-CONICET, Colón 4302, B7608FDQ Mar del Plata, Argentine; 2 Biology Department, FCEyN, National University of Mar del Plata, Funes 3250, B7602AYJ Mar del Plata, Argentine; 3 CIT Villa María–CONICET, Carlos Pellegrini 211, 5900 Villa María, Argentine; 4 UTN-FRVM, Av. Universidad 450, 5900 Villa María, Argentine; 5 Marine and Coastal Research Institute (IIMyC), Biology Department, FCEyN, National University of Mar del Plata-CONICET, Funes 3250, B7602AYJ Mar del Plata, Argentine

**Keywords:** Osseointegration, Zirconium implants, Anodization, Histology, Histomorphometry

## Abstract

Since surface plays a key role in bioactivity, the response of the host to the biomaterial will determine the success or failure of the prosthesis. The purpose of this study is to make an exhaustive analysis of the histological and histochemical characteristics of new bone tissue around Zr implants anodized at 60 V (Zr60) supported by histomorphometric methods in a rat model. Fibrous tissue was observed around the control implants (Zr0) and osteoblasts were identified on the trabeculae close to the implantation site that showed typical cytological characteristics of active secretory cells, regardless of the surface condition. The histomorphometrical analysis revealed a significant increase in cancellous bone volume, trabecular thickness and in trabecular number together with a decrease in trabecular separation facing Zr60. TRAP staining showed that there was a relative increase in the number of osteoclasts for Zr60. In addition, a larger number of osteoclast with a greater number of nuclei were detected in the tibiae for Zr60. This research demonstrated that the new bone microarchitecture in contact with Zr60 is able to improve the early stages of the osseointegration process and consequently the primary stability of implants which is a crucial factor to reduce recovery time for patients.

## Introduction

The main challenge in bone implant technology is the development of materials that enhance early phases of osseointegration and ensure long-term stability of its physical and mechanical properties (Misra et al. 2009).

In recent years, zirconium (Zr) has been proposed as a promising material for use in biomedical implants, since it has high values of bending strength and fracture toughness and excellent resistance to corrosion and wear (Zander and Köster 2004). These advantageous properties, determined in part by the presence of a thin native oxide film (ZrO_2_) on the surface of this pure material, result in a marked biocompatibility and a favorable tissue response to the implanted material (Sollazzo et al. 2008; Gomez Sanchez et al. 2011). Based on these properties, Zr and Zr-based alloys have been proposed as potential candidate for the development of permanent metal prostheses for orthopedic surgeries (Branzoi et al. 2008; Farina et al. 2015).

Previous studies have shown that the osseointegration process involves a series of events at the cellular and extracellular levels that take place at the material–biological host interface (Anselme et al. 2010; Guadarrama Bello et al. 2017). Therefore, the chemical and topographic characteristics of the material’s surface are critical for the substrate–tissue interaction and, together, they have a great influence on the cellular behavior (Woodruff et al. 2007; Dos Santos et al. 2009). Consequently, a large number of investigations proposed the use of surface modification techniques to generate a protective and bioactive film on metallic materials (Rouahi et al. 2006; Eliaz et al. 2009; Goriainov et al. 2014).

Among the different strategies for surface modification, anodizing is an economic method that has been widely used to obtain a uniform surface oxide layer with the aim of improving the performance of implants (Sul 2003; Yang et al. 2004; Gomez Sanchez et al. 2013). In particular, we have previously applied this electrochemical treatment on pure Zr, analyzing in detail its resulting surface characteristics (Gomez Sanchez et al. 2011; Sanchez et al. 2013). In these previous studies, it has been demonstrated that anodization in phosphoric acid modifies the topography and increases the ZrO_2_ thickness together with the incorporation of P into the oxide structure. This, in turn, can induce the precipitation of Ca–P compounds on zirconium oxide surface and, all together, improve its corrosion resistance. In addition, in vitro tests have demonstrated that the ZrO_2_ surface created by anodic oxidation at 60 V enhances cell spreading and metabolic activity (Katunar et al. 2017). In this study, the bone tissue integration around control and anodized Zr implants at 30 V and 60 V has already been examined in a rat femur model after 15 and 30 days of implantation. The results showed that Zr anodized at 60 V showed the highest bone thickness after 15 days of implantation giving a hint in the improvement of the primary stabilization of the implant when compared with the one anodized at 30 V and the control. After 30 days of implantation, the bone thickness for the three surfaces under study was comparable (Katunar et al. 2017).

Since anodizing treatment at 60 V has shown to induce an effective improvement in the osseointegration of Zr implants, the understanding of the morphofunctional characteristics of the bone tissue generated around anodized Zr implants deserves further investigation. Therefore, the aim of the present study is to make an exhaustive analysis of the histological and histochemical characteristics of bone tissue around control and zirconium implants anodized at 60 V, complemented by histomorphometric methods.

## Materials and methods

### Implants and surface treatment

Commercially pure zirconium cylinders (99.5% Roberto Cordes S.A., Argentina) of 40–50 mm length and 1 mm diameter were used for the in vivo tests. Two surface conditions were compared: as-received pure zirconium (Zr0, control) and zirconium anodized at a constant potential of 60 V (Zr60) during 60 min in 1 mol L^−1^ H_3_PO_4_. All samples were mechanically polished with 600 grit emery paper, degreased with ethanol and rinsed with deionized water. The sample conditioning and oxide growth details have been previously reported (Gomez Sanchez et al. 2011).

### In vivo studies

Twelve-week-old male WKAH/Hok rats (n = 12) weighing 300–330 g were used in this study. The animals were divided into two groups for each type of surface treatment: control (Zr0) and anodized (Zr60). All animals were housed in a temperature-controlled room with a 12 h alternating light–dark cycle and were given water and food ad libitum throughout the study. All the experiments were approved by the Bioethics Committee HIEMI-HIGA (Mar del Plata, October 2011).

### Surgical procedure

Rats were anesthetized with ketamine/xylazine (10 mg/kg; 10 mg/kg) according to their weight. The animals were placed in a supine position and the implantation place was cleaned with povidone iodine. The insertion was done in the proximal site of the tibia using a low-speed hand drill with a 0.15 diameter bur. Anodized and control implants were placed into the marrow channel, resulting in two implants per rat. Conventional nylon suture was used for closing the wounds. Rats were X-rayed after surgery to ensure the implant is in the proper location. Animals received tramadol (75 mg/kg) intraperitoneally as an analgesic until 72 h after surgery.

### Histological analysis

Fifteen days after implantation, rats with control (Zr0) and anodized (Zr60) implants (all individuals from different litters) were deeply anesthetized with ketamine/xylazine (100 mg/kg, 10 mg/kg) and sacrificed with 10% pentobarbital sodium (4 mg/100 g). After euthanasia, the proximal epiphysis of both tibiae was fixed in 10% phosphate-buffered formaldehyde and decalcified in 10% EDTA pH 7.4. Then, samples were dehydrated through ascending ethanol concentrations and embedded in paraffin wax (Krmpotic et al. 2015). Longitudinal 6-μm-thick sections of proximal tibiae were obtained with a rotary microtome (Leitz 1512, Germany). Sections were stained with hematoxylin–eosin (H–E) and Masson–Goldner ‘s trichrome for histomorphometrical and histological analysis. In addition, tartrate-resistant acid phosphatase (TRAP) staining was performed to evaluate osteoclastic activity. Micrographs were taken with an Olympus microscope CH30 (Olympus; Tokyo, Japan).

### Bone histomorphometry

Samples of 12-week-old male WKAH/Hok rats were used to perform the morphometric study, in which at least five peri-implant regions of 2 mm^2^ (ROI) per section were measured. Images (40X magnification) were captured using a digital video camera (Olympus DP731, Tokyo, Japan) mounted on a microscope (Olympus BX530, Tokyo, Japan) and processed using digital image analysis software (ImagePro Plus v6.3, Media Cybernetics, Bethesda, MD, USA).

The following measurements were performed: (1) total tissue volume, TV; (2) trabecular bone volume, BV; and (3) trabecular bone surface, BS. With these values, histomorphometric indices were quantitatively evaluated, including (1) bone volume, BV/TV (%) = [BV × 100/TV]; (2) trabecular thickness, Tb.Th (µm) = [2/(BS/BV)]; (3) trabecular number, Tb.N (1/mm) = [(BV/TV)/(Tb.Th)]; and (4) trabecular separation, Tb.Sp (µm) = [(1/Tb.N) − Tb.Th] following ASBMR standards as described previously (Compston et al. 2018).

### TRAP staining

Tartrate-resistant acid phosphatase (TRAP) staining was used to identify osteoclasts in histological sections. TRAP activity was detected by the azo-dye method. Briefly, the staining solution was prepared with Fast Garnet GBC salt (0.9 mmol L^−1^), 1.25 mg/mL Naphthol AS-BI phosphoric acid in dimethyl formamide, and L(þ)-tartaric acid (0.67 mol L^−1^) all diluted in sodium acetate buffer (2.5 mol L^−1^, pH 5.2). Deparaffinized sections were incubated in the solution for 40 min at 37 °C and then counter-stained with color fast kit (Biopack).

Quantitative histological evaluations of osteoclasts were then performed. TRAP-positive cells with multiple nuclei (> 3) and located on the bone surface or in Howship’s lacunae were identified as osteoclasts. The number of osteoclasts was counted using ImagePro plus 6.0 software (Media Cybernetics, USA). Osteoclast counts were standardized by dividing the number of osteoclasts by tissue volume.

### Statistical analysis

In this study, data were analyzed with Graph Pad In Stat version 3.00 (Graph Pad software) and all results were expressed as mean ± S.E.M. Histomorphometrical statistical analysis were performed by a parametrical assays Student’s *t* test, while osteoclast statistical analyses were performed by a non-parametric assay (Mann–Whitney test). All statistical analyses were considered significant when *p* value < 0.05.

## Results

### Clinical observations

The animals recovered mobility, feed functions and health perfectly well after the surgery and neither signs of infection nor inflammation were noted upon clinical examination during the experiment.

### Histological characterization

**Fig. 1:**
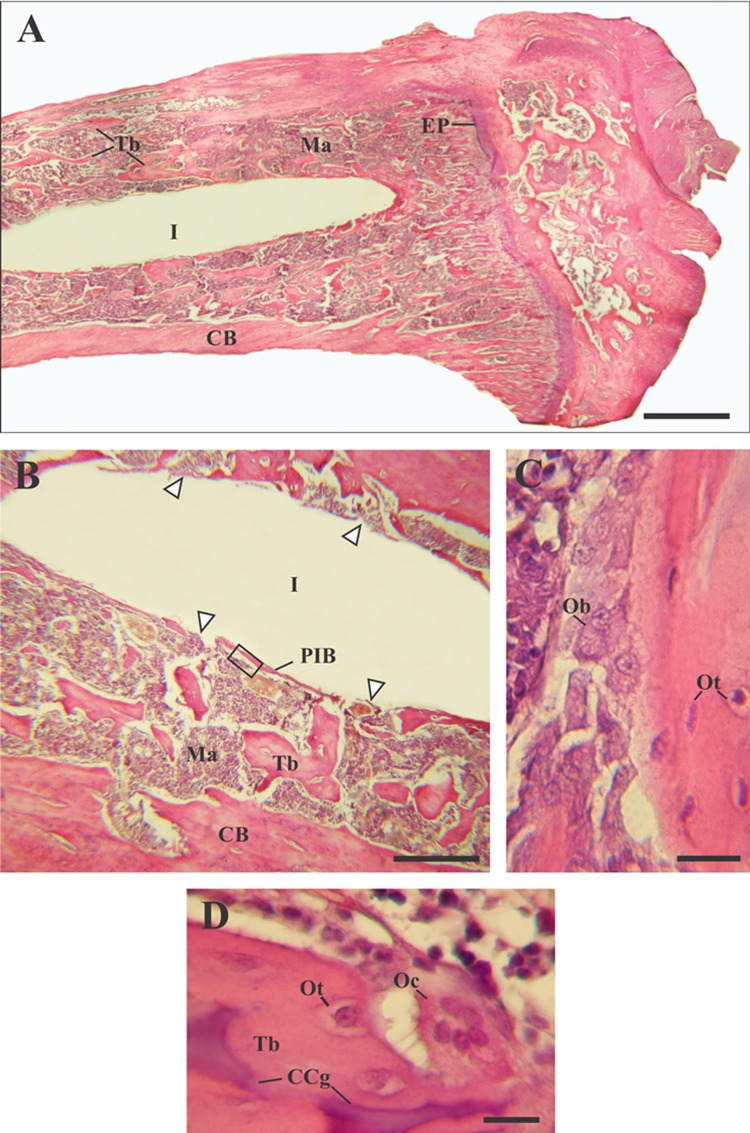
Light micrographs of new bone generated around control implants (Zr0), H–E. **a** Photomicrography of the rat tibia longitudinal section. **b** Details of the trabecular bone tissue surrounding the implantation site. **c** Enlargement of image B (black inset) showing active osteoblasts of the peri-implant bone tissue (PIB). **d** Photomicrography of an osteoclast on mixed trabeculae. Arrowheads, regions where PIB was not observed; *CB* compact bone; *CCg* calcified cartilage; *EP* epiphyseal plate; *I* region where the implant was located; *Ma* bone marrow; *Ob* osteoblasts; *Oc* osteoclasts; *Ot* osteocytes; *Tb* trabeculae. Scale bar: 170 μm (**a**); 50 μm (**b**); 12 μm (**c**, **d**)

**Fig. 2:**
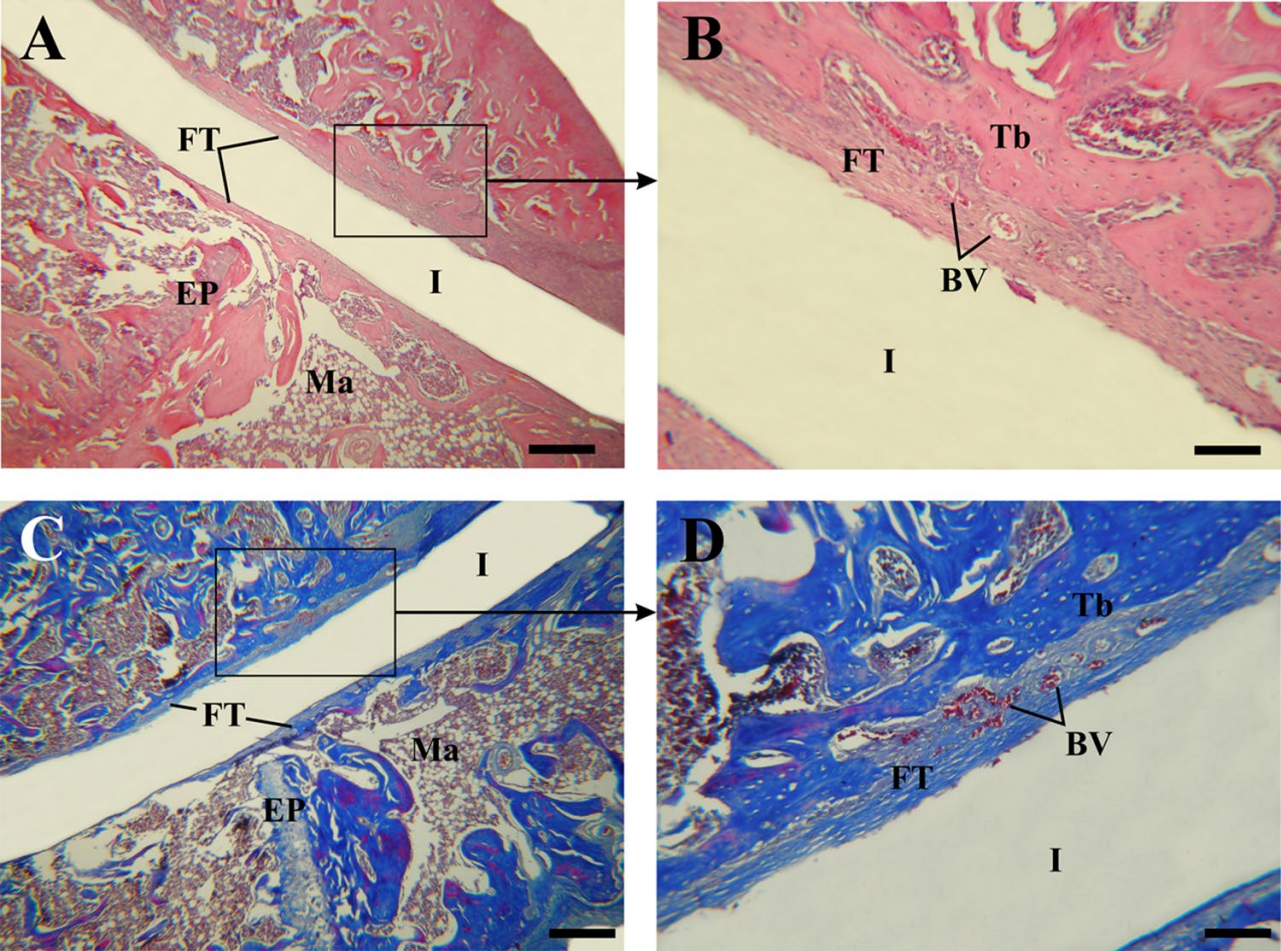
Light micrographs of fibrous tissue generated around control implants (Zr0). **a** Microphotograph showing the implantation site encapsulated by connective tissue. H–E. **b** Enlargement of (**a**) (black inset), H–E. **c** Photomicrography showing fibrous collagen tissue surrounds the implantation site, Masson–Goldner’s trichrome. **d** Enlargement of (**c**) (black inset), Masson–Goldner’s trichrome. *Ma* bone marrow; *EP* epiphyseal plate; *FT* fibrous tissue; *I* region where the implant was located; *Tb* trabeculae; *BV* blood vessel. Scale bar: 160 μm (**a**); 45 μm (**b**); 170 μm (**c**); 50 μm (**d**)

**Fig. 3:**
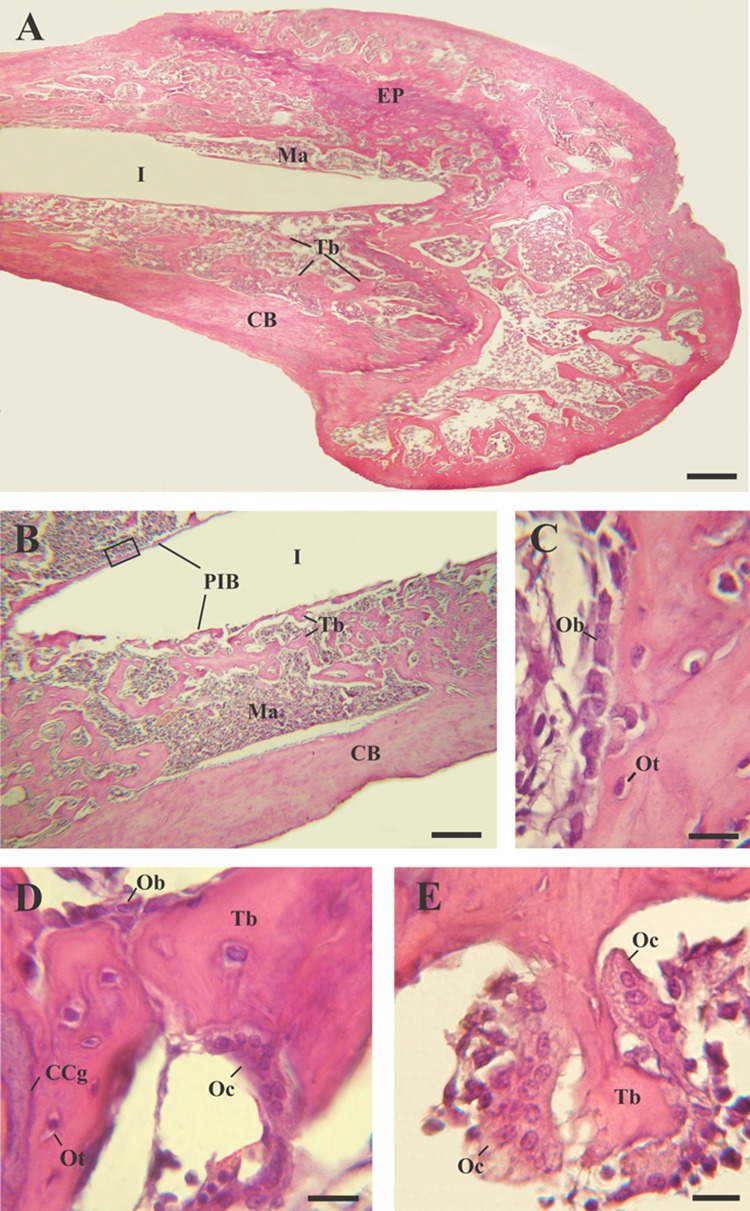
Light micrographs of new bone generated around anodized implants (Zr60), H–E. **a** Microphotography of the rat tibia longitudinal section. **b** Details of the trabecular bone tissue surrounding the implantation site. **c** Enlargement of image B (black inset) showing active osteoblasts on the left surface of the peri-implant bone tissue (PIB). **d** Photomicrography of an osteoclast on mixed trabeculae. **e** Details of osteoclasts on a bone trabeculae. *CB* compact bone; *CCg* calcified cartilage, *EP* epiphyseal plate; *I* region where the implant was located; *Ma* bone marrow; *Ob* osteoblasts; *Oc* osteoclasts; *Ot* osteocytes; *PIB*, *Tb* trabeculae. Scale bar: 165 μm (**a**); 50 μm (**b**); 10 μm (**c**–**e**)

At 15 days after implantation, tibiae of both experimental groups (Zr0 and Zr60) presented morphological characteristics typical of long bones. The free surface of the epiphysis was covered by articular cartilage in which chondrocytes in lacunae (called chondroplast) and an extracellular vascular matrix were identified. The bone underlying the articular cartilage was spongy and presented numerous trabeculae separated by medullary spaces. The diaphysis presented a wide medullary cavity (implantation site), surrounded by trabecular bone and covered by an outer layer of cortical bone. Since the implants were removed during the histological processing of the samples, the region they occupied was observed in the microphotographs as a white space inside the medullary cavity (Figs. 1a, b; 2; 3a, b).

The histological analysis showed modifications in the characteristics of the trabecular bone tissue surrounding the implants depending on the material’s surface in close contact with the bone. Around Zr0 implants, a predominance of bone marrow was observed over the trabecular bone tissue (Fig. 1a, b). The trabeculae were distinguished as individual spicules of bone surrounded by a large amount of bone marrow (Fig. 1b). On the other hand, two tibiae belonging to the control group presented peri-implant fibrous tissue with abundant collagen fibers distributed parallel to the main axis of the implant (Fig. 2a, c). This tissue was located between the trabecular bone tissue and the implantation’s area and was characterized by being highly vascularized (Fig. 2b, d). In both samples, the implantation site was extended to the proximal epiphysis, traversing the epiphyseal disc (Fig. 2a, c). In contrast, the bone tissue surrounding the Zr60 implants presented a large number of anastomosing trabeculae (Fig. 3a, b). Unlike the control samples, no peri-implant fibrous tissue was detected in the tibiae with anodized implant (Figs. 3a, b;5a). It is worth noting that neither of the two experimental groups (control and treatment) showed signs of inflammatory infiltrate in the implantation area.

**Fig. 4:**
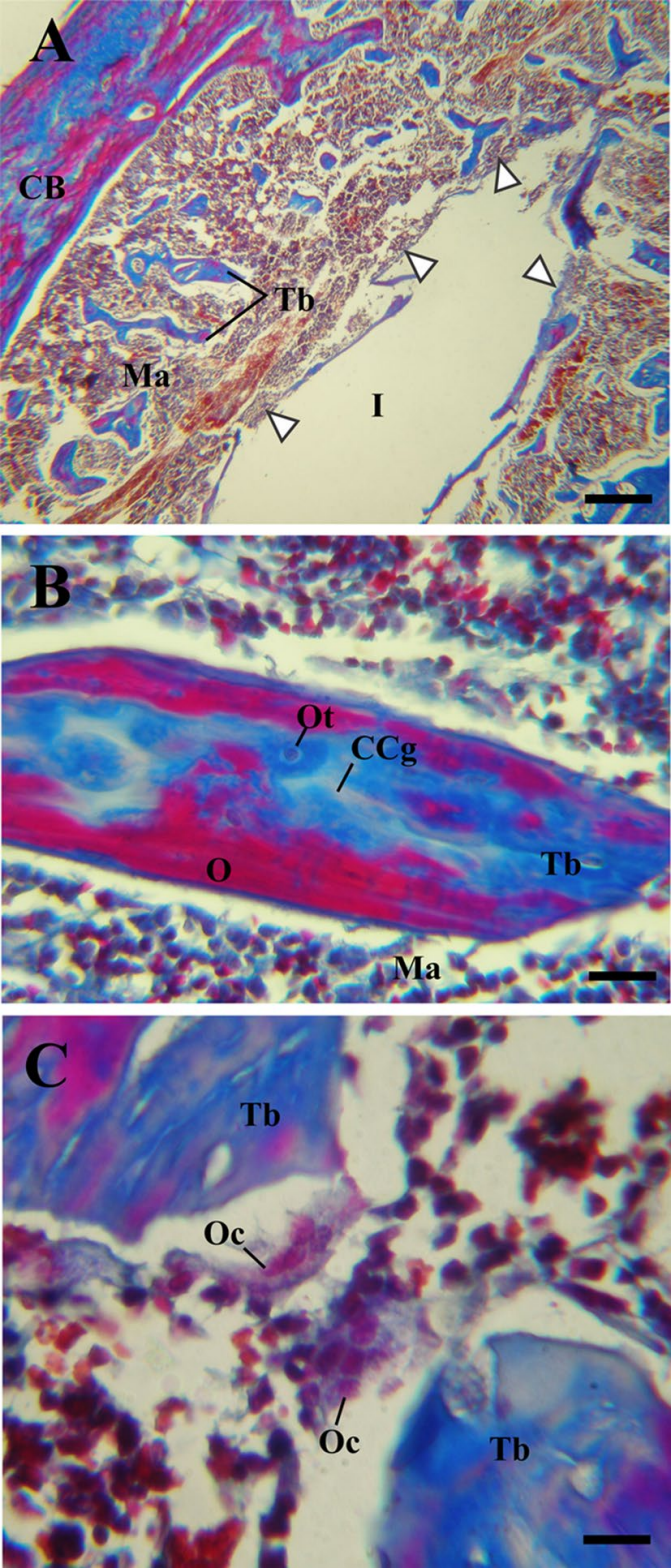
Light micrographs of new bone characteristics around control implants (Zr0), Masson–Goldner’s trichrome. **a** Photomicrography showing the peri-implant bone tissue. **b** Details of mixed trabeculae in the peri-implant region. **c** Details of mature osteoclasts. Arrow heads, regions where the peri-implant bone tissue was not observed; *Ma* bone marrow; *CB* compact bone; *CCg* calcified cartilage; *I* region where the implant was located; *O* osteoid; *Oc* osteoclasts; *Ot* osteocytes; *Tb* bone trabeculae. Scale bar: 175 μm (**a**); 45 μm (**b**); 12 μm (**c**)

At higher magnification, the histological analysis confirmed that all tibiae analyzed presented laminar bone in close contact with the implantation site, with the exception of those that exhibited fibrous tissue around control implants. Because of its location, this tissue was denominated as peri-implant bone tissue (PIB) (Figs. 1b, 3b). In control zirconium implants, the PIB was formed by thin isolated trabeculae separated by abundant bone marrow (Figs. 1b, 4a). In contrast, the PIB found in the tibiae Zr60 presented a greater thickness in comparison with the control group and, in addition, it was observed covering, in a continuous way, the peri-implant region (Fig. 3b). In both types of samples (Zr0 and Zr60), the PIB was coated with osteoblasts, indicating an active bone formation in the peri-implant region (Figs. 1c, 3c).

**Fig. 5:**
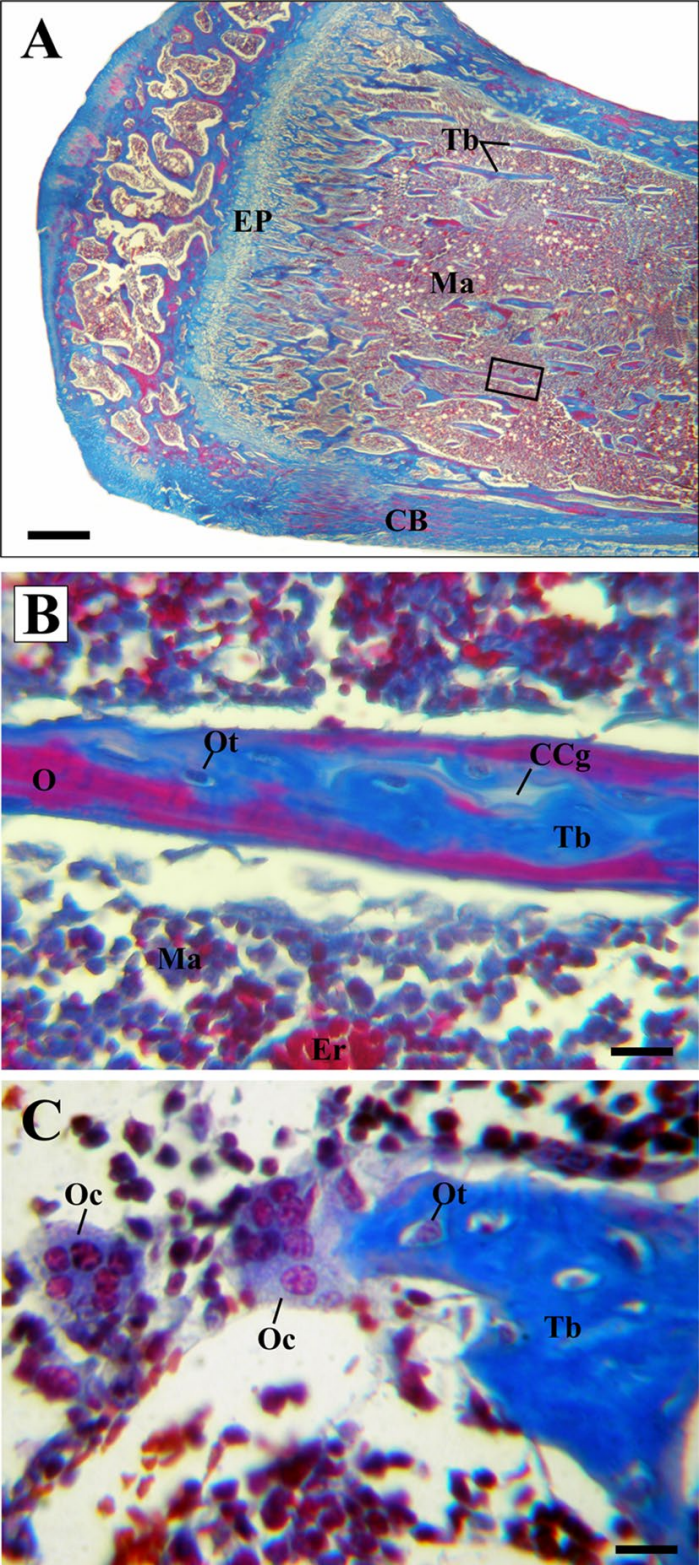
Light micrographs of new bone characteristics around anodized implants (Zr60), Masson–Goldner’s trichrome. E(**a**) piphysis. **b** Details of mixed trabeculae in the peri-implant region. **c** Morphological identification of different cell types in the peri-implant region. *Ma* bone marrow; *CB* compact bone; *CCg* calcified cartilage; *EP* epiphyseal plate; *Er* erythrocytes; *O* osteoid; *Oc* osteoclasts; *Ot* osteocytes; *Tb* bone trabeculae. Scale bar: 175 μm (**a**); 45 μm (**b**); 12 μm (**c**)

Regarding the characteristic cells of the bone tissue, no differences were found between the cytology of the osteocytes and the osteoblasts present in the tibiae with Zr0 and Zr60 implants. In both experimental groups, osteocytes were identified by their location in lacunae or osteoplasts within the bone matrix (Figs. 1c, d; 3c, d; 4b; 5b); and the osteoblasts were recognized as having a cuboid or polyhedral shape, basophilic cytoplasm and by following a mono-stratified linear distribution around the trabecular surface (Figs. 1c, 3c, d). In both experimental groups, the osteoclasts were always located in areas close to the trabecular bone and were identified by their acidophilic cytoplasm and the presence of multiple loose chromatin nuclei. These cells presented certain cytological differences between the tibiae with control and anodized implants, being larger and presenting a greater number of nuclei in the tibiae Zr60 (Figs. 1d; 3d, e; 4c; 5c).

The main histological differences of the bone tissue generated around Zr0 and Zr60 implants are detailed in Table 1.

**Table 1:** Main histological findings of new bone generated around control (Zr0) and anodised (Zr60) implants

Histological characteristics	Implants	
	Zr0	Zr60
Trabecular bone	A predominance of bone marrow was observed over thin isolated trabecular bone tissue facing the implant	The bone tissue surrounding implants presented a large number of anastomosing trabeculae
Fibrous tissue	Some implants were encapsulated by connective tissue	No soft tissues were detected around the implantation area
Peri-implant bone tissue (PIB)	PIB was formed by thin isolated trabeculae separated by abundant bone marrow	PIB was continuos and covered the peri-implant region
Osteoclast morphology	Multinucleated acidophilic cells in intimate contact with the trabecular surface	Larger number of osteoclasts and greater number of nuclei (compared to the control samples)

### Bone histomorphometry

Hematoxylin and eosin images depicted the bone–implant interface and trabecular topography among rats in contact with Zr0 and Zr60 implants. The quantitative histomorphometry evaluation provided detailed information about trabecular parameters around the Zr60 implants (Table 2). Fifteen days after implantation new bone facing Zr60 implants showed a significant increase in the BV/TV, Tb.N and TbTh compared to control implants followed by a significant reduction in TB.Sp (*p* < 0.05). Lower BV/TV in Zr0 compared with Zr60 implants was due to a decrease in trabecular thickness and number and an increase in trabecular separation.

**Table 2:** Histomorphometric and TRAP analysis of new bone generated around control (Zr0) and anodised (Zr60) implants

Implant	BV/TV (%)	Tb.Th (µm)	Tb.N° (1/µm)	Tb.Sp (µm)	Oc N°/mm^2^
Zr0	20.77 ± 0.92	41.77 ± 1.49	5.03 ± 0.28	165.50 ± 10.96	3.34 ± 0.31
Zr60	34.66 ± 1.09^*^	51.55 ± 1.49^*^	6.75 ± 0.16^*^	98.53 ± 3.38^*^	3.91 ± 0.30

Bone volume fraction (BV/TV); trabecular thickness (Tb.Th); trabecular number (Tb.N°); trabecular separation (Tb.Sp); osteoclast number (Oc N°).Data are expressed as mean ± SEM (parametrical assays Student’s *t* test for histomorphometrical analysis and non-parametric assay Mann–Whitney test for osteoclast analyses)

*p < 0.05 vs Zr0

### TRAP

**Fig. 6:**
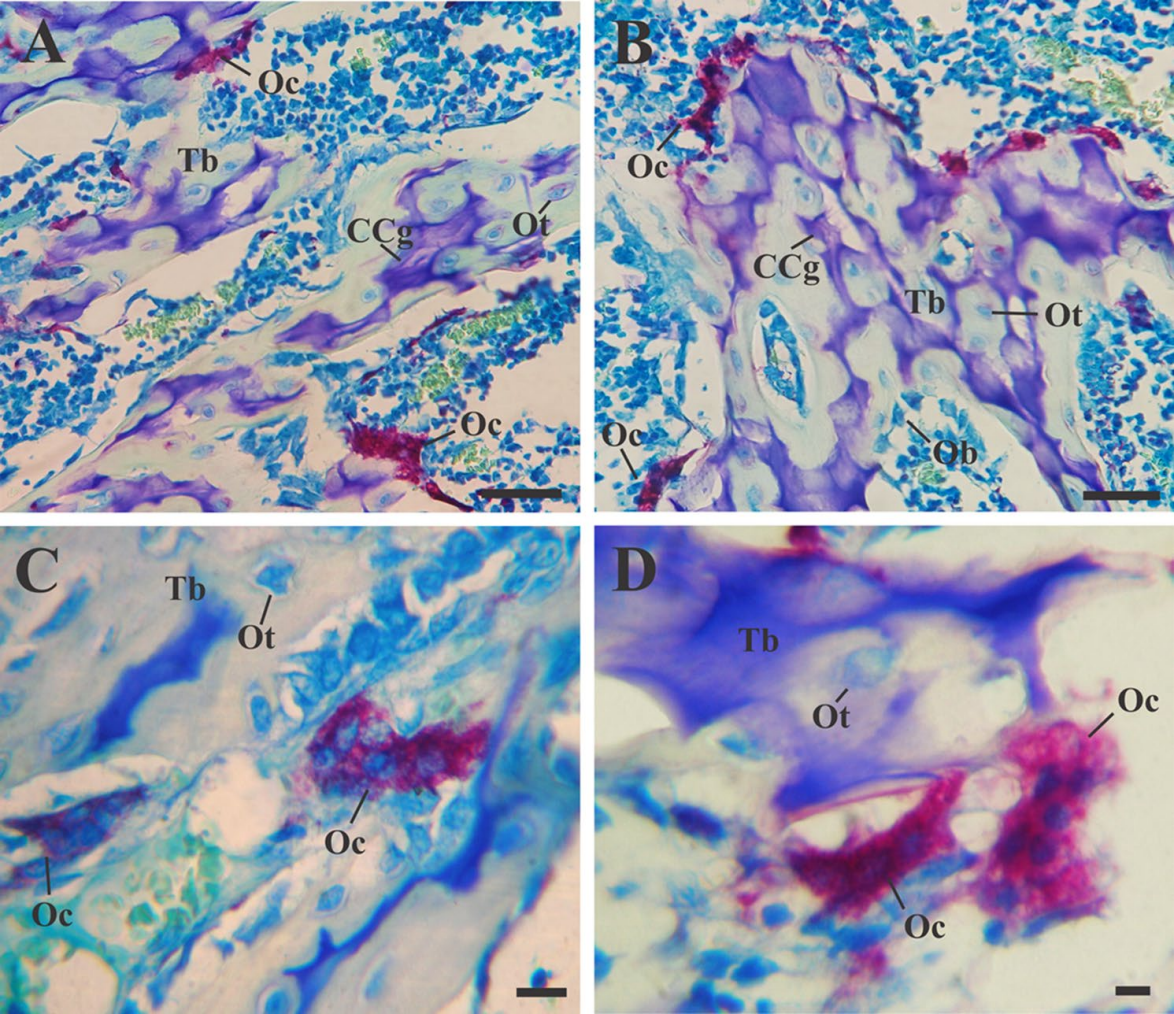
TRAP staining for osteoclast identification around Zr0 (A, C) and Zr60 (B, D) implants. **a**, **b** TRAP-positive cells were stained red and observed to be closely related to the bone surface. **c**, **d** Higher magnification of Figures **a** and **b** showing multinucleated TRAP-positive cells. *CCg* calcified cartilage; *Ob* osteoblasts; *Oc* osteoclasts; *Ot* osteocytes; *Tb* trabeculae. Scale bar: 45 μm (**a**, **b**); 9 μm (**c**, **d**)

The enzymatic activity of tartrate-resistant acid phosphatase (TRAP) was determined in situ as an osteoclast marker to evaluate the bone resorption process of the bone tissue surrounding the Zr0 and Zr60 implants. The TRAP-positive cells showed a reddish granular cytoplasmic staining and the characteristic phenotype of the osteoclasts (Fig. 6). The statistical analysis revealed that there are no significant differences between the number of osteoclasts present in the tibiae Zr0 and Zr60 at 15 days after the implantation surgery (Table 2). However, the size and the number of this cell type per unit area showed a general increasing tendency in tibiae with anodized implants compared with the control tibiae.

## Discussion

A promising osseointegration of bone implants is essential for safe implant functionality and the prevention of implant failure in future (Cooper 1998, 2000). It is well-known that implant surface plays a key role in the rate and in the success of implant osseointegration process (Cooper 1998; Nanci et al. 1998; Isa et al. 2006; Totea et al. 2014; Pellegrini et al. 2018). Surface characteristics such as chemistry (Meirelles et al. 2008), roughness (Deligianni et al. 2001), wettability (Lampin et al. 1997) and surface energy (Biggs et al. 2007) are some of the most critical factors that affect cell and tissue–materials interaction (Das et al. 2009). As the surface is the only region in contact with host bone tissue, many attempts have been made to modify the surface properties improving the host tissue integration and mechanical fixation as well. Therefore, several strategies have been carried out to optimize implant surfaces for improving the different stages of the osseointegration process. Various methods have been developed to obtain different implant surfaces characteristics such as plasma spray, grid blasting, acid etching and anodization (Sul et al. 2002; Rupp et al. 2006; Le Guéhennec et al. 2007; Reyes et al. 2007; Mendonça et al. 2008; Milošev 2010; Novaes et al. 2010; Gomez Sanchez et al. 2011, 2013), that produce mainly modification in the topography and in the chemical composition of the surface.

Bone, as all connective tissues, is composed of cells and an extracellular matrix mainly comprised of a collagen type I network impregnated with hydroxyapatite mineral crystals. The cortical bone is compact, enervated and vascularized and is present in the epiphyses of long bones and coats the bones of the body, giving rigidity and strength. Otherwise, the trabecular part of bone is formed by a network of trabeculae, which confers resistance to compression. Bone is constantly remodeled by the dual action of two cell types, osteoclasts and osteoblasts. While the osteoclasts are involved in the resorb activity, the osteoblasts have the function of generating new bone. This complex process is required for the development and the correct maintenance of the bone tissue, taking into account that the modeling at bone surface has a higher rate in trabecular in comparison to cortical bone (Kini and Nandeesh 2012; Kohli et al. 2018). In consequence, the analysis of bone microarchitecture, the texture of the osteoid matrix and the presence of changes in the mineralization and remodeling process of both surface and interstitial layers are crucial parameters to analyze bone metabolism. Therefore, Compston et al. (2018) evaluated the importance of the study of bone microarchitecture and proposed a set of stereological techniques for its measurement. These techniques are used worldwide giving rise to systems of classification, rules and standardization of measurement units allowing the histomorphometric study to transform into a useful tool for monitoring bone processes (Arlot et al. 2008). Histological and histochemical techniques allow relating the structural characteristics of tissues and organs with the composition and localization of specific molecules. In particular, the goal of staining histology slides for osseointegration studies is to visualize both the inflammatory reaction and the healing responses within or in contact with the biomaterial evaluated (i.e., the classic hematoxylin and eosin [H&E] stain for histology) as well as describing and evaluating the new bone formation (i.e., employing special stains such as Stevenel’s blue or Goldner’s trichrome). In some cases, additional special stains reactions are necessary in some studies to fully evaluate a targeted marker or response such as the tartrate-resistant acid phosphatase (TRAP) staining.

Osseointegration has been defined as a crucial factor for implant success and can be defined as a direct structural and functional connection between living bone and the implant surface (Albrektsson and Johansson 2001). A failure in the osseointegration process is largely related to the encapsulation of the implant by connective tissue that prevents colonization of the bone cells and isolates the implant from the host tissue (Civantos et al. 2017). It is well-known that after implantation, an immune system response is triggered, with macrophage-specific polarization to pro-inflammatory (M1) or anti-inflammatory (M2) phenotypes depending on several factors, including biomaterial surface properties (Jetten et al. 2014). There is recent in vitro evidence that the surface modification of zirconium implants by anodization treatment at 60 V can regulate the M1/M2 macrophage balance to the M2 anti-inflammatory phenotype, this being an optimal immune response to successful implant integration (Katunar et al. 2017). In agreement with this finding, in the present study, only fibrous tissue was observed around some of the Zr0 implants, while all implants with anodized treatment exhibited peri-implant bone tissue without inflammatory signs. Moreover, osteoblasts were identified on the trabeculae close to the implantation site that showed typical cytological characteristics of active secretory cells, regardless of the surface condition (Zr0 and Zr60). These observations also are in line with previous in vivo studies, which have shown a significant increase in the mineral apposition rate around Zr60 implants compared to the control condition (Katunar et al. 2014).

The histomorphometry analysis developed in this work could contribute to the evaluation of new bone microarchitecture in close contact with the anodized zirconium implants 15 days after implantation. The parameters, BIC (bone–implant contact), BV/TV, bone mineral density, BA/TA (bone area fraction), mean trabecular thickness, mean trabecular number and mean trabecular separation have been used to quantify the response at the bone–implant interface (Vandamme et al. 2010; Steiner et al. 2015). The results of the present study showed that the histomorphometric results revealed a significant increase in cancellous bone volume (BV/TV,  %), which is a percentage of the total marrow cavity that is occupied by cancellous bone (both mineralized and non-mineralized) facing Zr60 implant and a significant increase in trabecular thickness (Tb.Th) and in trabecular number (Tb.N). In addition, a significant decrease in trabecular separation was detected (Tb.Sp) suggesting that the new bone microarchitecture in contact with anodized implants at 60 V may improve the osseointegration process and consequently the anchorage of the implants. Our results are in line with the results found by Cheng et al. (2016), who reported a simple strategy to modify the surface of titanium (Ti) implants by loading them with Sr and Ag to impart osteogenic and anti-bacterial properties after nanotubular anodization treatment. Their assays suggested that the modified implants would be highly effective for promoting bone healing and quick in vivo osseointegration in a rat femoral defect model when compared to simple Ti implants at 4 and 6 weeks after surgery. Additional, He et al. demonstrated by histomorphometry assays and micro-CT images that the parameters BV/TV and BA/TA were significantly higher in plasma electrolytic oxidation (PEO)-treated Ti surfaces than in raw titanium surfaces 2 weeks after surgery in a rat femur implant model (He et al. 2017). They found that modified titanium surfaces showed better bioactivity compared with pure titanium surfaces and there was also a significant time-specific and site-specific difference between the PEO and raw Ti groups for BV/TV 15 days after implantation.

As skeletal mass homeostasis is maintained by a local balance between osteoclastic bone resorption and osteoblastic activities resulting in bone remodeling (Furuya et al. 2018; Kohli et al. 2018; Iaquinta et al. 2019), TRAP staining was employed to study osteoclastic activity and distribution of osteoclast at the implantation site. Our results showed that there is a relative increase in the number of osteoclasts (N.Oc) present in tibiae with Zr60 implants compared to the control group, although there were no significant differences between the two groups. This tendency to increase N.Oc in tibiae Zr60 is consistent with previous results found in vitro by Katunar et al., where it was demonstrated that Zr60 produces a greater recruitment and differentiation to mature osteoclasts of murine macrophage cell line RAW 264.7 on the surface of the anodized material compared to the control group (Katunar et al. 2017). In addition, the histological analysis allowed detecting some cytological differences between the osteoclasts present in tibiae with Zr0 and Zr60 implants, being larger and presenting a greater number of nuclei in the tibiae in contact with Zr60. This histological description is in agreement with the morphological characteristics described previously in vitro, in which osteoclast had several nuclei and many lysosomal vesicles (Katunar et al. 2017). The results of the present study, together with our previous in vitro assay, provide evidence that the anodizing process on Zr assesses a substrate modification that allows osteoclast differentiation through RANK–RANKL pathway and osteoclast-mediated bone resorption. These findings are relevant given that, although osteoclast activity is generally associated with bone resorption it has been demonstrated that they are able to secrete mediators which can induce the migration and osteogenic differentiation of mesenchymal stem cells to the site of bone remodeling (Pederson et al. 2008; Kreja et al. 2010; Gamblin et al. 2014; Kusumbe and Adams 2014). In this way, the transition from bone resorption to formation during the remodeling process is mediated by osteoclast-derived coupling factors. Based on the results shown, it can be noticed that Zr60 presents an increased osteoclast differentiation capacity when compared with Zr0, which is currently known to be necessary for normal bone formation.

## Conclusion

This study showed that Zr anodized at 60 V is able to promote a significant increase in cancellous bone volume, in trabecular thickness and in trabecular number with the consequent decrease in trabecular separation when compared with the control after 15 days of implantation. These facts suggest that the new bone microarchitecture in contact with anodized implants at 60 V is able to improve the osseointegration process and consequently the primary stability of implants which is a key factor to reduce patient’s mobility and lead to quicker recovery.
